# The importance of inter‐individual variation in predicting species' responses to global change drivers

**DOI:** 10.1002/ece3.4810

**Published:** 2019-03-28

**Authors:** Ella Guscelli, John I. Spicer, Piero Calosi

**Affiliations:** ^1^ Département de Biologie, Chimie et Géographie Université du Québec à Rimouski Rimouski Québec Canada; ^2^ Marine Biology and Ecology Research Centre, School of Biological and Marine Sciences University of Plymouth Plymouth UK

**Keywords:** acid–base regulation, acid–base status, individual variation, ocean acidification, ocean warming, phenotypic variation

## Abstract

Inter‐individual variation in phenotypic traits has long been considered as “noise” rather than meaningful phenotypic variation, with biological studies almost exclusively generating and reporting average responses for populations and species’ average responses. Here, we compare the use of an individual approach in the investigation of extracellular acid–base regulation by the purple sea urchin *Paracentrotus lividus *challenged with elevated *p*CO_2_ and temperature conditions, with a more traditional approach which generates and formally compares mean values. We detected a high level of inter‐individual variation in acid–base regulation parameters both within and between treatments. Comparing individual and mean values for the first (apparent) dissociation constant of the coelomic fluid for individual sea urchins resulted in substantially different (calculated) acid–base parameters, and models with stronger statistical support. While the approach using means showed that coelomic *p*CO_2_ was influenced by seawater *p*CO_2_ and temperature combined, the individual approach indicated that it was in fact seawater temperature in isolation that had a significant effect on coelomic *p*CO_2_. On the other hand, coelomic [HCO_3_^−^] appeared to be primarily affected by seawater *p*CO_2_, and less by seawater temperature, irrespective of the approach adopted. As a consequence, we suggest that individual variation in physiological traits needs to be considered, and where appropriate taken into account, in global change biology studies. It could be argued that an approach reliant on mean values is a “procedural error.” It produces an artefact, that is, a population's mean phenotype. While this may allow us to conduct relatively simple statistical analyses, it will not in all cases reflect, or take into account, the degree of (physiological) diversity present in natural populations.

## 1 INTRODUCTION

Real individuals are unique combination of traits, some above and some below average. It is time to re‐cognize the uniqueness of the individual and to turn it to our advantage as biologists. (Bennett, 1987)

Darwin (1859) introduced the notion that variation is a prerequisite for natural selection to operate. More than a century later, Bennett (1987) suggested that the variability in organismal responses, due to the inter‐individual variation in natural populations or species, should not be written off as “noise,” but instead could provide new powerful insights into how organisms function and have evolved. And yet historically, and even currently, biological studies have largely focused on mean organismal responses, a method that can significantly underestimate the importance of the individual and its genetic and phenotypic characteristics (Aldrich, 1975; Bennett, 1987; Spicer & Gaston, 1999).

The relative paucity of studies that take an inter‐individual approach most likely biases our perception and understanding of organisms’ physiological processes. The mean values we commonly generate and use in our analyses can be considered useful abstractions or even artefacts constructed to aid analysis or interpret (i.e., simplifying) our perception of nature. While convenient and powerful, a mean does not capture the level of phenotypic variation that exists in natural populations. This emphasis on mean values has been termed “the tyranny of the Golden Mean” (Bennett, 1987). In turns, the broad acceptance of the utilization of this approach has led us to consider values of central tendency being more important and meaningful than the data used to generate it (Spicer & Gaston, 1999).

Inter‐individual variation plays a critical role in determining activity patterns (Dias, Granadeiro, & Catry, 2013; Vardanis, Klaassen, Strandberg, & Alerstam, 2011), food preferences, foraging activity (Arnold, 1981; Newsome et al., 2009), and metabolic responses (Aldrich, 1975; Hayes, Garland, & Dohm, 1992; Spicer & Baden, 2001; Vézina, Speakman, & Williams, 2006), all of which can scale up to community responses (Bolnick et al., 2011; Hanski & Singer, 2001; Spicer & Gaston, 1999) and ecosystem functions (Cianciaruso, Batalha, Gaston, & Petchey, 2009). Studying inter‐individual variation has also been insightful in investigating potential impacts of climate change (e.g., Schlegel, Havenhand, Gillings, & Williamson, 2012; Ellis et al., 2017). However, little is known about the implications of individual variation within physiological systems in defining species responses within the context of global change, (Small, Calosi, Boothroyd, Widdicombe, & Spicer, 2015; Walther, Anger, & Pörtner, 2010) and shift in species biogeography (Deutsch, Ferrel, Seibel, Pörtner, & Huey, 2015; Parmesan & Yohe, 2003; Pörtner & Knust, 2007).

It is common practice to use constants derived from one species or population, and expressed as a mean value, in the calculation of physicochemical characteristics of an organism. This could be because it has not been possible to derive that constant for the individual or species of interest, there was a desire to minimize “nuisance” inter‐individual variation, or because the statistical analysis is more straightforward if mean data were used. However, it is possible that an individual approach could produce different, and potentially more accurate, outcomes of environmental challenge, compared with more traditional mean‐based approaches.

On a different but related note, it is often assumed that constants derived from one system or species are equally applicable to another. For example, the mean dissociation constant (pKʹ_1_) for hemolymph from the shore crab *Carcinus maenas* has often been used to calculate acid–base parameters (e.g., *p*CO_2_ and [HCO_3_^−^]) for other species when empirical values are not available (e.g., Calosi, Rastrick, et al., 2013; Miles, Widdicombe, Spicer, & Hall‐Spencer, 2007; Spicer, Raffo, & Widdicombe, 2007). However, the efficacy of doing so has never been formally assessed.

The ongoing increase in human activities since the industrial revolution has resulted in an accumulation of greenhouse gases in the atmosphere (IPCC, 2013), leading to a net increase in subsurface ocean temperature, a phenomenon termed ocean warming (OW). Changes in temperature have significant physiological implications for biological life, particularly ectothermic organisms (Pörtner & Knust, 2007), ultimately leading to changes in their geographical distribution and shifts in food webs (e.g., Stachowicz, Terwin, Whitlatch, & Osman, 2002; Perry, Low, Ellis, & Reynolds, 2005). In parallel, the increase in atmospheric CO_2_ is in part, buffered, as approx. 30% of emitted CO_2_ is stored in the world's oceans (Sabine & Feely, 2007; Sabine et al., 2004). This has resulted in an increase in bicarbonate ion ([HCO_3_^−^]), and a decrease in seawater carbonate concentration ([CO_3_^2−^]) and pH (Caldeira & Wickett, 2003; Cao and Caldeira, 2008). This phenomenon, termed ocean acidification (OA), poses a challenge to many marine organisms with a calcareous skeleton, such as corals, mollusks, echinoderms, and crustaceans (Binyon, 1972; Fabry, Seibel, Feely, & Orr, 2008; Vinogradov, 1953). In particular, OA has been shown to have potential negative impacts on marine organisms by altering functions such as acid–base balance and mineralization (Findlay et al., 2011; Melzner et al., 2009; Orr et al., 2005; Pörtner, 2008; Widdicombe & Spicer, 2008). Perhaps the greatest challenge faced by marine organisms is the interaction between OW and OA, which may produce additive, synergistic, or negative effects on the development (e.g., Arnberg et al., 2013; Gianguzza et al., 2013; Padilla‐Gamino, Kelly, Evans, & Hofmann, 2013; Wangensteen, Dupont, Casties, Turon, & Palacin, 2013), physiology (Byrne, 2012; Melatunan, Calosi, Rundle, Moody, & Widdicombe, 2011; Small et al., 2015; Todgham & Stillman, 2013), and life history (e.g., Byrne, 2011; Kroeker et al., 2013; Pistevos, Calosi, Widdicombe, & Bishop, 2011; Melatunan, Calosi, Rundle, Widdicombe, & Moody, 2013; Small et al., 2015) of marine ectotherms.

The importance of maintaining a healthy acid–base status is critical as it ensures successful intracellular enzymatic processes, such as protein synthesis and ATP production (Grainger, Winkler, Shen, & Steinhardt, 1979; Walsh & Milligan, 1989). This results in positive growth and reproductive investment, ultimately determining the fitness of an individual. This said, the significance of inter‐individual variation on acid–base responses to perturbation has rarely been considered while investigating the impact of OW and OA on marine ectotherms (although cf. Pistevos et al., 2011; Schlegel et al., 2012; Calosi, Turner, et al., 2013). Furthermore, to our knowledge, there is no published work where individual‐level variation in the acid–base balance of ectotherms has been investigated within the context of the ongoing global change.

Consequently, the aims of this study are threefold. First, to characterize the level of inter‐individuals variation in acid–base parameters of an ectotherm exposed to OW and OA conditions. Secondly, to assess the difference and the implications of using an individual‐based approach, compared with an approach using mean values for pK_1_ʹ from the same species, when investigating the acid–base responses of a species exposed to global change challenges. And finally to assess the difference, and the implications, of using an individual‐based approach or an approach using mean values for pK_1_ʹ from the same species, compared with an approach using mean values for pK_1_ʹ from a different species. The second and third aims will provide insight into understanding the significance of inter‐individual variation in physiological responses and processes, ultimately used to predict species responses to global change drivers.

Sea urchins (Echinoidea) are considered to be among some of the most vulnerable groups to OA as they show limited capacity to buffer changes in pH (Miles et al., 2007; Spicer & Widdicombe, 2012; Spicer, Widdicombe, Needham, & Berge, 2011; Stumpp et al., 2013; Stumpp, Hu, et al., 2012; Stumpp, Trübenbach, Brennecke, Hu, & Melzner, 2012). Urchins’ relatively poor regulatory ability emerges when they are exposed to fluctuation in seawater temperature and chemistry parameters that affect their ability to maintain their acid–base status (Farmanfarmaian, 1966). Nevertheless, there is growing evidence that they are able to compensate respiratory acidosis *via *buffering with bicarbonate ions ([HCO_3_^−^]) internal acidosis (Stumpp, Trübenbach, et al., 2012; Walsh & Milligan, 1989) and possess some ability for ionic regulation when exposed to seawater fluctuations (Calosi, Rastrick, et al., 2013; Freire, Santos, & Vidolin, 2011; Vidolin, Santos‐Gouvea, & Freire, 2007). Consequently, the purple sea urchin Paracentrotus lividus (Binyon, 1972) was chosen as an ideal model for our study.

To achieve our aims, we exposed adult individuals of *P. lividus* to combinations of three seawater temperatures and two seawater *p*CO_2_ in an orthogonal experimental design. First, we measured the pH and total carbon dioxide content (TCO_2_) of coelomic fluid from each individual tested. We then calculated the first apparent dissociation constant (pKʹ_1_) for coelomic fluid from that individual. We then calculated the first apparent dissociation constant (pK_1_ʹ) (a) for coelomic fluid for each individual urchin, (b) as a mean value derived from values for a number of urchins. These pK_1_ʹ values were then used to generate individual and mean acid–base parameters of the coelomic fluid. These same parameters were also calculated for urchin coelomic fluid using pK_1_ʹ values derived from crab hemolymph. We then qualitatively compared the outputs of the three approaches, in order to test whether mean approaches are representative of the outcome of the analyses considering inter‐individual variation in acid–base ability shown by individuals of *P. lividus*.

## 2 MATERIALS AND METHODS

### 2.1 Urchin collection, transport, and maintenance

Adult individuals of the purple sea urchin *P. lividus* were collected from the Dunmanus Bay aquaculture facility (Country Cork, Ireland—51°33ʹ7.2ʺN, 9°43ʹ12ʺW) on 20/01/2014. Urchins were packed in plastic bags (approx. 40 indiv. per bag, vol. = 15 L) each filled with oxygen saturated seawater (*S* = 33). Bags were placed in a polystyrene box with ice, and shipped overnight to Bristol (UK) and then transported within 3 hr by car to the Marine Biology and Ecology Research Centre (MBERC) Laboratory, University of Plymouth (UK). Immediately upon arrival, sea urchins were transferred to aquaria (vol. = 300 L, approx. 40 indiv. per aquarium), supplied with water (*S* = 33, *T* = 15°C) from a recirculating seawater system connected to a filter (2213 External Filter, Eheim GmbH & Co., Deizisau, Germany) all of which was housed within a controlled‐light and room temperature at L:D 12 hr:12 hr. Here, urchins were maintained for 3 days before use in any experiment, in order to recover and fed seaweed *Laminaria digitata* ((Huds.) Lamouroux, 1813) ad libitum.

### 2.2 Experimental design, procedure, and preparation

To determine the combined effect of elevated seawater temperature and partial pressure of CO_2_ (*p*CO_2_) on coelomic fluid parameters of urchins, we employed a quadratic experimental design with two levels of seawater *p*CO_2_ and three levels of seawater temperature. Individual urchins were therefore exposed to one of six possible combinations of present‐day seawater *p*CO_2_ (≈390 µatm) and predicted seawater *p*CO_2_ value for the year 2,100 (≈1,000 µatm—IPCC, 2013), and one of three temperatures (10, 15, 20°C) selected to mimic a range of temperature representative of the thermal range this species currently experiences in the Atlantic and in future due to the ongoing warming (IPCC, 2013). Consequently, experimental conditions were as follows: “control” (10°C + *p*CO_2_ 380 µatm), “elevated *p*CO_2_” (10°C + *p*CO_2_ 1,000 µatm), “elevated temperature” (15°C + *p*CO_2 _380 µatm), “elevated temperature and *p*CO_2_” (15°C + *p*CO_2_ 1,000 µatm), “extreme temperature” (20°C + *p*CO_2_ 380 µatm), and “extreme temperature and elevated *p*CO_2_” (20°C + *p*CO_2_ 1,000 µatm).

Approx. 23 individuals were individually placed in labeled mesh hand‐made cages (mesh size 1 × 1 cm, Cage vol. = 0.5 L) and each cage assigned haphazardly to each of the six treatments. Urchins were fed ad libitium during the exposure period but were starved 24 hr prior to coelomic fluid sampling to avoid postprandial increases in metabolic activity.

Finally, urchins were observed every day before and after a water change, as well as following sampling, to visually determine their health conditions and survival.

### 2.3 Experimental setup, environmental monitoring, and carbonate system characterization

Urchins were held in large trays (vol. = 300 L) filled with seawater aspirated with either untreated air (*p*CO_2_ ≈ 380 µatm) or CO_2_‐enriched air (*p*CO_2_ ≈ 1,000 µatm). Inside the trays, the desired seawater temperature was maintained using chillers (L‐350, Guangdong Boyu Group & CO., Guangdong, China) and aquarium stick heaters (3614, Eheim, Deizisau, Germany) in combination. To aid water mixing, each tray was fitted with a submersible pump (Koralia Nano Evolution 900, HYDOR USA Inc., Sacramento, CA USA). Water changes were performed daily to maintain good water quality. For details on the environmental monitoring and carbonate system characterization, see the dedicated section and the Figure S1 in the Appendix S1.

### 2.4 Perivisceral coelomic fluid sampling and analyses

To avoid the negative effects of multiple intrusive (i.e., with a needle) sampling, a single coelomic fluid was taken at the end of the exposure period (7 days). While obtaining multiple samples from the same individual is good practice to avoid bias from intra‐individual variation on the characterization of inter‐individual variation (Bennett, 1987), intrusive sampling of the coelom activates an immune response (Smith et al., 2010) by introducing bacteria and other pathogens: Changes in cellular immune condition co‐occurred with changes in extracellular acid–base balance of the green sea urchin *Strongylocentrotus droebachiensis* were reported by Dupont and Thorndyke (2012). Therefore, we had to assume that such acid–base responses were repeatable and did so on the basis of the acid–base individual‐level responses of *S. droebachiensis* exposed to stable seawater conditions in a previous study (see Appendix S1: Figure S4). Here, sea urchins displayed relatively stable and “consistent” coelomic fluid pH and bicarbonate concentrations when these parameters were measured repeatedly over time (see Appendix S1: Figure S4).

Perivisceral coelomic fluid (vol. = 500 µl) was extracted anaerobically at day 7 from each individual using a gas‐tight syringe (500 µl, 1750 RN, Hamilton Bonaduz, Switzerland) while positioning the urchin ventral side uppermost, submerged just below to the water surface. The needle of the syringe was carefully inserted at an angle of approx. 45° and to a depth of 10 mm through the soft membrane surrounding the Aristotle's lantern directly into the urchins’ perivisceral coelom. A second sample was obtained from the main coelomic cavity by positioning the needle at about 90° relative to the oral surface and deeply inserting it into the individuals’ main extracellular space (as described in Calosi, Rastrick, et al., 2013). Great care was taken to avoid damaging the gut and gonads, and thereby contaminating coelomic fluid.

Measuring coelomic fluid carbon dioxide and pH was carried out using well‐established methods (Donohue et al., 2012; Marchant, Calosi, & Spicer, 2010; Miles et al., 2007; Rastrick et al., 2014; Small, Calosi, White, Spicer, & Widdicombe, 2010; Spicer et al., 2007). To determine coelomic fluid TCO_2_, 50 µl of fluid was introduced anaerobically into a previously calibrated CO_2_ analyzer (965D, Ciba Corning Diagnostic Cor., Cambridge, MA, USA) less than 30 s after sampling. The pH of the coelomic fluid (pH_cf_) was measured, within 60 s of extraction. The sample was placed in a microcentrifuge tube (1.5 ml, Fisherbrand, Thermo Fisher Scientific Inc.) and a micro‐pH electrode (MI‐413, Microelectrodes, Bedford, MA, USA) immersed in the fluid. The electrode was coupled to a calibrated pH meter (Five Easy, Mettler Toledo). TCO_2 _and pH_cf_ measures were performed at the respective temperature of incubation according to the treatment at which the urchin was exposed in order to maintain constant environmental conditions and avoid animals stress.

Unused samples of coelomic fluid were frozen at *T* = −20°C in a microcentrifuge tube (1.5 ml) and subsequently used to determine individuals’ non‐bicarbonate buffer (NBB) line (see below).

### 2.5 Determination of individuals’ pK_1cF _values, *p*CO_2cf_, and [HCO_3_^−^]_cf_

The first (apparent) dissociation constants for the coelomic fluid (pKʹ_1cf_) from *P. lividus* were determined for individual urchins and used to calculate coelomic fluid *p*CO_2 _(*p*CO_2cf_) and coelomic fluid [HCO_3_^−^]_cf_ ([HCO_3_^−^]_cf_). pKʹ_1cf_ were estimated for each coelomic fluid sample tonometered (400 µl in gassing chamber, Sci‐Glass Consultancy, Devon, UK) against a range of CO_2_ tensions (0.04 to 1.01 kPa roughly equivalent to 0.3 to 7.6 mmHg) supplied by precision gas mixing pumps (Wösthoff, Bochum, FRG). Using tonometers coelomic fluid samples were maintained at the environmental temperature the urchin was exposed to (*T* = 10, 15 and 20°C). Both TCO_2cf_ and pH_cf_ were measured, as described above, at each CO_2_ tension.

The functional pKʹ_1_ value for the coelomic fluid from each individual urchin was then calculated over the pH range obtained, using the Henderson–Hasselbalch equation in the form:

(1) $${\text{pK}}_{1\text{cf}}^{\prime }=\text{pH}-\log\left[\left({\text{TCO}}_{2}-\alpha p{\text{CO}}_{2}\right)/\alpha p{\text{CO}}_{2}\right]$$

where *α* is the solubility coefficient of CO_2_ in *Carcinus maenas *hemolymph (0.058 mmol L^−1^ mm Hg^−1^, 0.050 mmol L^−1^ mm Hg^−1^, and 0.043 mmol L^−1^ mm Hg^−1^, at 10, 15, and 20°C, respectively: Truchot, 1976). The equation for the in vitro NBB line was calculated by plotting pH_cf_ against corresponding [HCO_3_^−^]_cf_, the latter value calculated using the equation:

(2) $$\left[{\text{HCO}}_{3}^{-}\right]={\text{TCO}}_{2}-\alpha p{\text{CO}}_{2}$$

Values for in vivo *p*CO_2_ were calculated from direct measurements of TCO_2cf_ and pH_cf_ from the same individual using the Henderson–Hasselbalch equation in the form:

(3) $$p{\text{CO}}_{2\text{cf}}={\text{TCO}}_{2}/\alpha \left(10^{\text{pH}-{\text{pK}}^{\prime }}+1\right)$$

The in vivo [HCO_3_^−^]_cf_ was then calculated using the Henderson–Hasselbalch equation in the form:

(4) $${\left[{\text{HCO}}_{3}^{-}\right]}_{\text{cf}}={\text{TCO}}_{2\text{cf}}-\left(\alpha p{\text{CO}}_{2\text{cf}}\right)$$

At high pH and low *p*CO_2_, carbamate concentration cannot be ignored (Truchot, 1976). Acidifying the environment resulted in an acidification of extracellular body fluids. Consequently, we estimated that any carbamate present would be in negligible quantities and so has not been calculated. Also, our calculated values for [HCO_3_^−^] may also include very small amounts of CO_2_ in other chemical forms.

### 2.6 Determination of mean *p*CO_2cf_ and [HCO_3_^−^]_cf_

To investigate the effect of adopting a “mean approach,” and to compare its outcome to the that for an individual approach, *p*CO_2cf_ and [HCO_3_^−^]_cf_ were calculated using the average value of individual sea urchins’ pKʹ_1cf_, as well as calculated using mean pKʹ_1cf_ values for *C. maenas*: 6.057, 6.029, and 6.000 at 10, 15, and 20°C (Truchot, 1976) as it is sometime customary, determined per each treatment condition in this study: see paragraph “Determination of individuals’ pK_1cf _values, *p*CO_2cf_ and [HCO_3_^−^]_cf_” and “Coelomic fluid *p*CO_2cf_ and [HCO_3_^−^]_cf_ determined using mean individual pKʹ_1_ for *Paracentrotus lividus*.”

### 2.7 Determination of key morphometric parameters

After the coelomic fluid was sampled, the height and diameter of the tests for each sea urchin were measured using a calliper (PD‐151, Pro's kit Industries Co., Ltd., Taiwan) and used to calculate the spheroidal volume (as in Calosi, Rastrick, et al., 2013). Finally, sea urchins were weighed with a digital high‐precision scale (PS‐200, Fisher Scientific Ltd., Corby, UK—0.1 mg accuracy).

### 2.8 Survival

No mortality was recorded in either the “elevated temperature” or the “extreme temperature” treatments, while among the other treatments between one and three urchins died during the experiment. No significant relationships between mortalities and seawater *p*CO_2_, temperature or their combination were detected (*χ* ^2^ = 2.25, *df* = 3, *p* = 0.522). In addition, survivors appeared in excellent health conditions, that is, none showed any noticeable loss in spines, and all responding actively by moving their spines and extruding their tube feet when coelomic fluid was sampled.

### 2.9 Statistical analysis

To investigate the effect of elevated *p*CO_2_, temperature and their interaction on urchins mortality and mean pH_cf_, TCO_2cf_, *p*CO_2cf_, and [HCO_3_^−^]_cf_, determined in the three different ways (a) individual pKʹ_1cf_ for *P. lividus *(from this study), (b) mean pKʹ_1cf_ for *P. lividus *(from this study), and (c) using mean pKʹ_1cf _for *C. maenas* (Truchot, 1976). We then used a two‐way analysis of covariance (ANCOVA) with individuals’ body volume (cm^3^), wet mass (g) as covariates. Finally, we compared the patterns of significance obtained from the three approached employed to explore whether substantial differences exist between these different approaches. The same analysis was conducted on the individual pKʹ_1cf_ to assess OW and OA combined effect on it. As preliminary analyses showed that covariates never exerted a significant effect on any of the traits investigated (maximum *F*_1,129_ = 1.021, *p* > 0.05), they were removed from further analyses and so we were able to use an ANOVA test. All data met the assumption for normality of distribution and homogeneity of the variance as untreated or Log_10_ transformed data, with the exception of pH_cf_. However, as log_10_ transformation was not beneficial for pH_cf_, and considering that our experimental design included six treatments with a minimum of 19 replicates per treatment per measurement, we assumed that the experimental design employed should be tolerant to deviation from the assumptions of normality and heteroscedasticity (Sokal & Rohlf, 1995; Underwood, 1997). In addition, for no variable investigated, a significant relationship between its unstandardized residuals and the factors investigated was found, indicating that as our experimental design was solid we could use row data for pH_cf_. Finally, pairwise comparisons were conducted using the estimated marginal means test with Fisher least significant difference (LSD) correction. All analyses were conducted using IBM SPSS Statistic 21.

## 3 RESULTS

### 3.1 Coelomic fluid pH_cf_, TCO_2cf_, and pKʹ_1cf_

Individual pH_cf_ values ranged between 6.05 and 8.07 (see Appendix S1: Figure S1a), and individual TCO_2cf_ values ranged between 1.4 and 8.8 mmol/L (see Appendix S1: Figure S1b).

**Figure 1:**
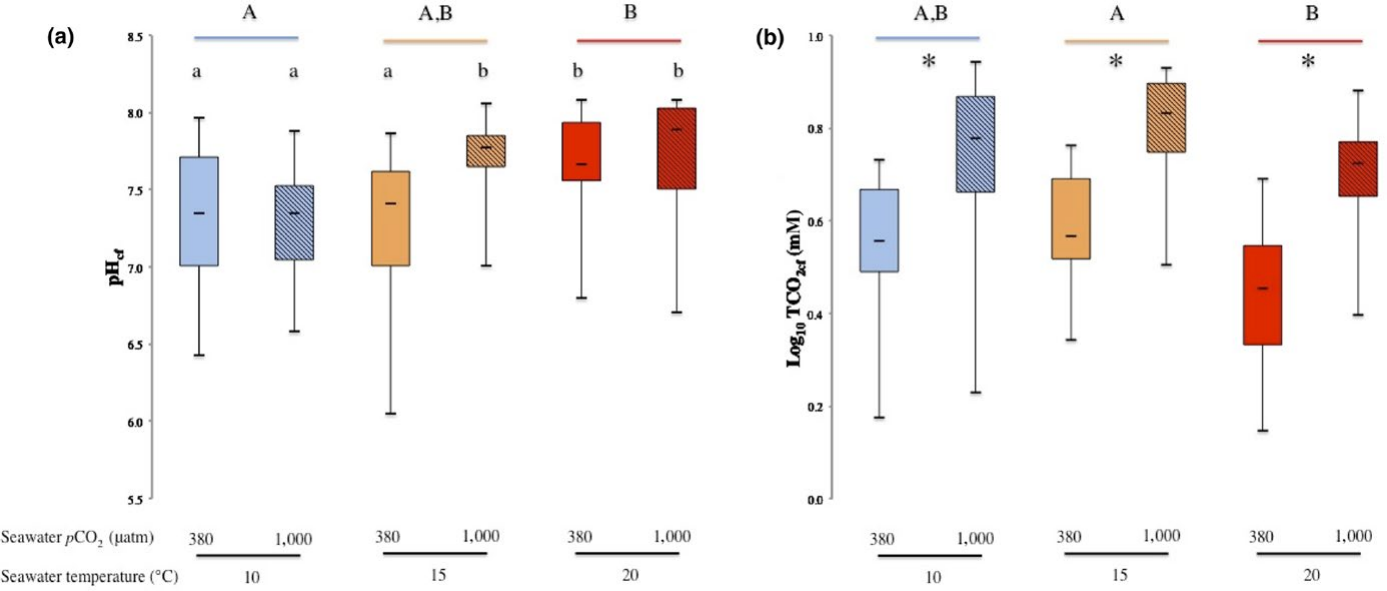
The effects of elevated seawater *p*CO_2_ and temperature on coelomic fluid (a) pH (pH_cf_) and (b) Log_10_ TCO_2_ (TCO_2cf_) of the sea urchin *Paracentrotus lividus*. Temperature treatments are indicated by blue, orange, and red colors for 10, 15, and 20°C, respectively. Ambient seawater *p*CO_2 _(≈300 µatm) and elevated seawater *p*CO_2 _(≈1,000 µatm) are indicated by plain and hatched box plot, respectively. Lower case letters identify significant differences (*p* < 0.05) between treatments. Capital letters identify significant differences (*p* < 0.05) between temperature treatments. Asterisks identify significant differences (*p* < 0.05) between *p*CO_2_ treatments at the same temperature treatment

The effects of exposure to seawater *p*CO_2_, temperature, and their combination on pH_cf_ and TCO_2cf_ are presented in Figure 1a,b, respectively.

Mean pH_cf_ was positively affected by elevated seawater *p*CO_2_ at 15°C, while there was no significant effect of this factor on mean pH_cf_ at 10 and 20°C, as indicated by a significant interaction between seawater *p*CO_2_ and temperature (see Table 1). In addition, seawater temperature in isolation had a significant effect on mean pH_cf_: mean pH_cf_ was greater in urchins kept at 20°C than those kept at 10°C. However, mean pH_cf _for urchins kept at 15°C was comparable to that of the other two temperatures tested (see Figure 1a).

**Table 1:** Results of ANOVAs investigating the effects of elevated *p*CO_2_ and temperature and their interaction on the coelomic fluid parameters of the sea urchin *Paracentrotus lividus*

Trait	Source	Sum of squares (type III)	*df*	MS	*F*	*p*
pH_cf_	*p*CO_2_		1	0.599	4.196	0.043
	Temperature		**2**	**1.561**	**10.929**	**<0.0001**
	Interaction		**2**	**0.701**	**4.91**	**0.009**
	Error		124	0.143		
	Total	7,334.798	130			
	Total correct	22.898	129			
Log_10_ TCO_2cf_	*p*CO_2_		**1**	**1.666**	**91.851**	**<0.0001**
	Temperature		**2**	**0.174**	**9.618**	**<0.0001**
	Interaction		2	0.01	0.548	0.58
	Error		124	0.018		
	Total	57.317	130			
	Total correct	4.268	129			
pKʹ_1cf_ (individual for *P. lividus*)	*p*CO_2_		1	0.011	0.164	0.686
	Temperature		**2**	**1.291**	**19.934**	**<0.0001**
	Interaction		2	0.043	0.66	0.519
	Error		124	0.065		
	Total	5,130.511	130			
	Total correct	10.738	129			
Log_10_ *p*CO_2cf_ (individual pKʹ_1cf_ for *P. lividus*)	*p*CO_2_		1	0.255	1.554	0.215
	Temperature		**2**	**4.165**	**25.337**	**<0.0001**
	Interaction		2	0.389	0.389	0.098
	Error		124	0.164		
	Total	35.487	130			
	Total correct	29.908	129			
Log_10_ [HCO_3_^−^]_cf_ (individual pKʹ_1cf_ for *P. lividus*)	*p*CO_2_		**1**	**1.693**	**91.736**	**<0.0001**
	Temperature		**2**	**0.167**	**9.042**	**<0.0001**
	Interaction		2	0.011	0.572	0.566
	Error		124	0.018		
	Total	56.401	130			
	Total correct	4.317	129			
Log_10_ *p*CO_2cf_ (mean pKʹ_1cf_ for *P. lividus*)	*p*CO_2_		1	0.28	2.466	0.119
	Temperature		**2**	**4.099**	**36.078**	**<0.0001**
	Interaction		**2**	**0.389**	**3.424**	**0.036**
	Error		124	0.114		
	Total	28.942	130			
	Total correct	23.520	129			
Log_10_ [HCO_3_^−^]_cf_ (mean pKʹ_1cf_ for *P. lividus*)	*p*CO_2_		**1**	**1.918**	**78.675**	**<0.0001**
	Temperature		**2**	**0.12**	**4.925**	**0.009**
	Interaction		2	0.021	0.861	0.425
	Error		124	0.024		
	Total	51.024	130			
	Total correct	5.196	129			
Log_10_ *p*CO_2cf_ (mean pKʹ_1cf_ for *C. maenas*)	*p*CO_2_		1	0.335	2.962	0.103
	Temperature		**2**	**1.559**	**12.523**	**<0.0001**
	Interaction		**2**	**0.576**	**4.623**	**0.012**
	Error		124	0.124		
	Total	44.090	130			
	Total correct	20.112	129			
Log_10_ [HCO_3_^−^]_cf _(mean pKʹ_1cf_ for *C. maenas*)	*p*CO_2_		**1**	**1.831**	**87.219**	**<0.0001**
	Temperature		**2**	**0.134**	**6.381**	**0.002**
	Interaction		2	0.014	0.681	0.508
	Error		124	0.021		
	Total	53.757	130			
	Total correct	4.712	129			

Note

cov: covariate; *df*: degrees of freedom; *F*: *F*‐ratio; MS: mean of square; *p*: probability level.

pH_cf_ and TCO_2cf_ were determined in vivo, while pKʹ_1cf_ in vitro and *p*CO_2cf_ and [HCO_3_^−^]_cf_ were calculated using: (a) individual pKʹ_1_ determined for *P. lividus*, (b) mean pKʹ_1_ for *P. lividus*, and (c) mean pKʹ_1_ for *C. maenas*. Significant *p*‐values are given in bold.

Mean TCO_2cf_ was significant greater at the higher seawater *p*CO_2_ conditions for all temperatures tested (see Table 1), while it was greater at 15°C and lower at 20°C, with seawater *p*CO_2_ and temperature having a significant positive and negative effect on mean TCO_2cf_, respectively (see Figure 1b and Table 1). Nonetheless, seawater *p*CO_2_ and temperature together had no significant effect on mean TCO_2cf_ (see Table 1).

**Figure 2:**
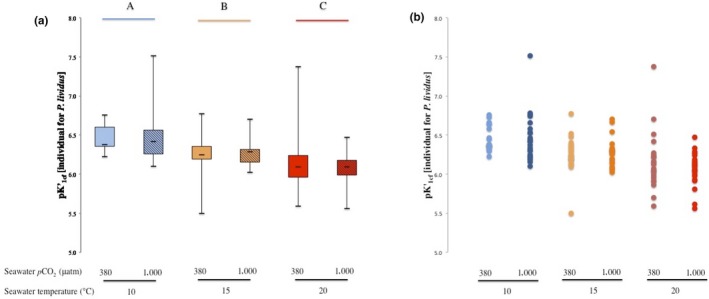
The effects of seawater *p*CO_2_ and temperature (a) on coelomic fluid pKʹ_1_ (pKʹ_1cf_) and (b) on inter‐individual variation of coelomic fluid pKʹ_1_ (pKʹ_1cf_) in the sea urchin *P. lividus*. Temperature treatments are indicated by blue, orange, and red colors for 10, 15, and 20°C, respectively. Ambient seawater *p*CO_2 _(≈300 µatm) and elevated seawater *p*CO_2 _(≈1,000 µatm) are indicated by (a) plain and hatched box plot, respectively and (b) by clear and darker colors, respectively. (a) Capital letters identify significant differences (*p* < 0.05) between temperature treatments and (b) dots identify individual measurements

Results for the analysis of individual pKʹ_1cf_ for *P. lividus* are presented in Figure 2a. Individual pKʹ_1cf_ values ranged between 5.50 and 7.51 (see Figure 2b).

Individual pKʹ_1cf_ values decreased significantly with increasing seawater temperatures (see Figure 2a and Table 1). There were no significant effects of seawater *p*CO_2_ on its own, or in combination with temperature (see Table 1).

### 3.2 Calculation of coelomic fluid *p*CO_2cf_ and [HCO_3_^−^]_cf_ determined using individual data for *P. lividus*

**Figure 3:**
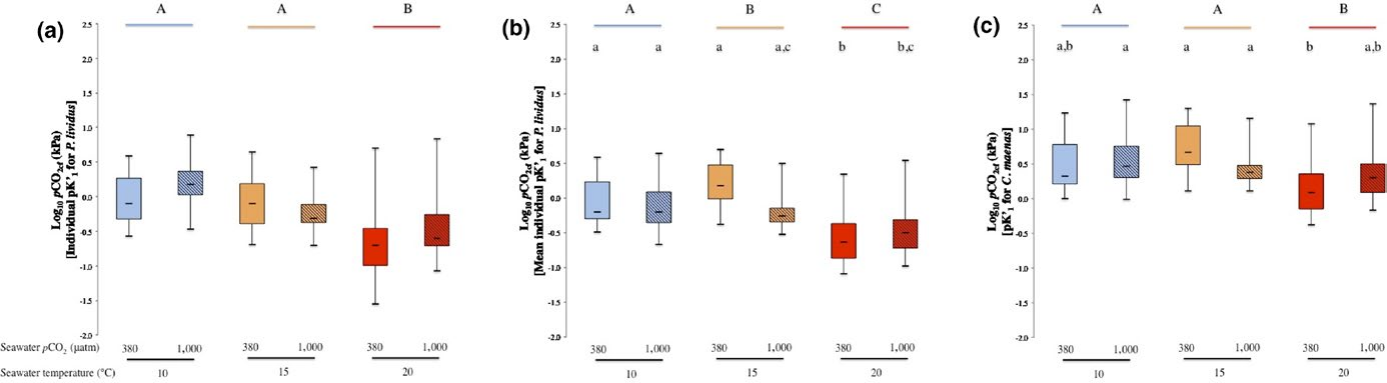
The effects of elevated seawater *p*CO_2_ and temperature on coelomic fluid Log_10_ *p*CO_2_ of the sea urchin *P. lividus*. *p*CO_2cf_ determined (a) using individual values of pKʹ_1_ for *P. lividus*, (b) using the mean individual pKʹ_1_ for *P. lividus,* and (c) using pKʹ_1_ for *Carcinus maenas*. Temperature treatments are indicated by blue, orange, and red colors for 10, 15, and 20°C, respectively. Ambient seawater *p*CO_2 _(≈300 µatm) and elevated seawater *p*CO_2 _(≈1,000 µatm) are indicated by plain and hatched box plot, respectively. Lower case letters identify significant differences (*p* < 0.05) between treatments. Capital letters identify significant differences (*p* < 0.05) between temperature treatments

**Figure 4:**
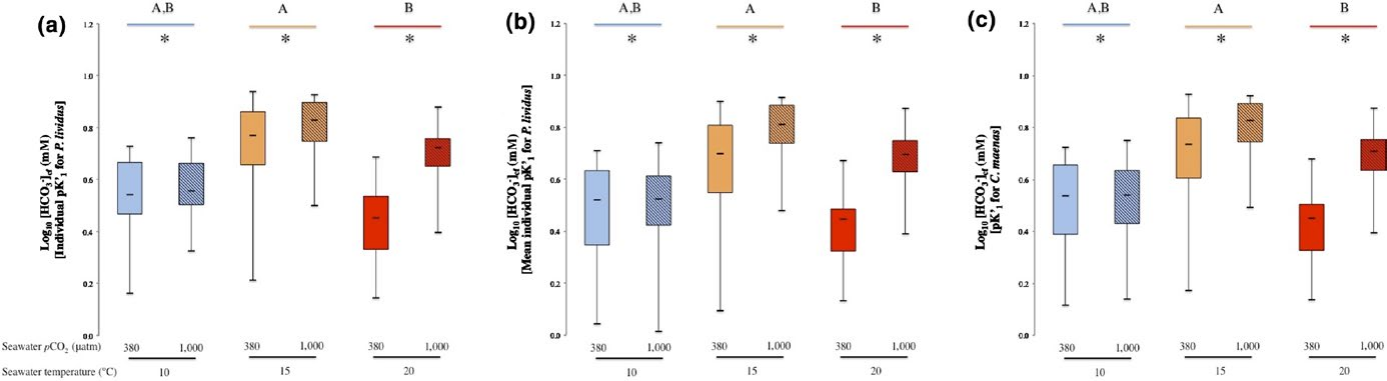
The effects of elevated seawater *p*CO_2_ and temperature on coelomic fluid Log_10_ [HCO_3_^−^] of the sea urchin *P. lividus*. [HCO_3_^−^]_cf_ determined using (a) individual values of pKʹ_1_ for *P. lividus*, (b) the mean individual pKʹ_1_ for *P. lividus*, and (c) pKʹ_1_ for *C. maenas*. Temperature treatments are identified by blue, orange, and red colors for 10, 15, and 20°C, respectively. Ambient seawater *p*CO_2 _(≈300 µatm) and elevated seawater *p*CO_2 _(≈1,000 µatm) are identified by plain and hatched box plot, respectively. Capital letters indicate significant differences (*p* < 0.05) between temperature treatments. Asterisks indicate significant differences (*p* < 0.05) between *p*CO_2_ treatments at the same temperature treatment

Results for the analysis of *p*CO_2cf_ and [HCO_3_^−^]_cf_ determined using individual data for *P. lividus* are presented in Figures 3a and 4a, respectively.

Individual *p*CO_2cf_ values ranged between 0.02 and 7.84 kPa (see Appendix S1: Figure S2a), and individual [HCO_3_^−^]_cf_ values ranged between 1.39 and 8.71 mmol/L (see Appendix S1: Figure S3a).

Seawater temperature had a negative effect on mean *p*CO_2cf_ for urchins kept at the highest temperature tested compared to those that experienced 10 and 15°C, and mean *p*CO_2cf_ values were significantly lower at 20°C (see Figure 3a and Table 1). Furthermore, neither seawater *p*CO_2_ on its own nor in combination with temperature had any significant effect on mean *p*CO_2cf_ (see Table 1).

There was a positive effect of seawater *p*CO_2_ on mean [HCO_3_^−^]_cf_ at all temperatures tested (see Table 1). Conversely, seawater temperature had a negative effect on mean [HCO_3_^−^]_cf_ between 15 and 20°C (see Figure 4a and Table 1), lower values being characteristic of the highest temperature tested (see Figure 4a). Additionally, seawater *p*CO_2_ effect was stronger than seawater temperature effect. (see *F*s in Table 1), while no effect of seawater *p*CO_2_ and temperature together was recorded (see Table 1).

### 3.3 Calculation of coelomic fluid *p*CO_2cf_ and [HCO_3_^−^]_cf_ using mean individual pKʹ_1_ for *P. lividus*

Mean individual pKʹ_1_ for *P. lividus* used to calculate coelomic fluid *p*CO_2cf_ and [HCO_3_^−^]_cf_ were 6.445, 6.256, and 6.158 for the present‐day seawater *p*CO_2_ treatment at 10, 15, and 20°C, respectively, and 6.466, 6.281, and 6.069 for the elevated seawater *p*CO_2_ treatment at 10, 15, and 20°C, respectively.

Results for the analysis of *p*CO_2cf_ and [HCO_3_^−^]_cf_ determined using the mean individual pKʹ_1_ for *P. lividus* are presented in Figures 3b and 4b, respectively.

Individual *p*CO_2cf_ values ranged between 0.08 and 4.99 kPa (see Appendix S1: Figure S2b), and individual [HCO_3_^−^]_cf_ values ranged between 1.03 and 8.23 mmol/L (see Appendix S1: Figure S3b).

Mean *p*CO_2cf_ was negatively affected at the highest seawater temperature, for the low seawater *p*CO_2_ treatment, compared with the other two seawater temperature tested (see Figure 3b), while there was a progressive negative effect on mean *p*CO_2cf_ from 10 to 20°C for the high seawater *p*CO_2_ treatment, as indicated by the presence of a significant interaction between seawater *p*CO_2_ and temperature (see Figure 3b and Table 1). Temperature in isolation had a negative stronger effect on mean *p*CO_2cf_ as there was a significant difference in mean *p*CO_2cf_ within all seawater temperature treatments (see Table 1), and mean values decreased with increasing seawater temperature (see Figure 3b).

There was a significant positive effect of seawater *p*CO_2_ on mean individual [HCO_3_^−^]_cf _at all temperature tested (see Table 1). On the contrary, seawater temperature in isolation had a negative effect on mean individual [HCO_3_^−^]_cf _between 15 and 20°C (see Table 1), lower mean values being found at the highest seawater temperature tested (see Figure 4b). However, seawater temperature effect in isolation was weaker than the effect of seawater *p*CO_2_ on its own (compare Fs Table 1). Additionally, no interaction between seawater *p*CO_2_ and temperature interaction was detected (see Table 1).

### 3.4 Calculation of coelomic fluid *p*CO_2cf_ and [HCO_3_^−^]_cf_ using mean pKʹ_1_ for *C. maenas*

Results for the analysis of *p*CO_2cf_ and [HCO_3_^−^]_cf_ determined using *C. maenas*’ pKʹ_1_ are presented in Figures 3c and 4c, , respectively.

Individual *p*CO_2cf_ values ranged between 0.05 and 3.51 kPa (see Appendix S1: Figure S2c), with the lowest mean values recorded at the highest seawater temperature (see Appendix S1: Figure S2c). Individual [HCO_3_^−^]_cf_ values ranged between 1.30 and 8.44 mmol/L (see Appendix S1: Figure S3c).

Mean *p*CO_2cf_ was reduced by increasing in temperature alone from 15 to 20°C, and only for the low seawater *p*CO_2_ treatments. In contrast, there was no significant effect of temperature while no effect of temperature on urchins exposed to high seawater *p*CO_2_ conditions, as shown by the significant interaction between seawater *p*CO_2 _and temperature (see Figure 3c and Table 1). Seawater temperature had a negative effect on mean *p*CO_2cf_ values between 15 and 20°C, being significantly lower at 20°C when compared to 10 and 15°C (see Figure 3c and Table 1).

No significant effect of the combination of altering seawater *p*CO_2 _and temperature on [HCO_3_^−^]_cf_ was detected (see Table 1), although seawater *p*CO_2 _on its own had a positive effect on mean [HCO_3_^−^]_cf_ at all seawater temperatures tested (see Table 1). That said seawater temperature had a negative effect on mean [HCO_3_^−^]_cf_ between 15 and 20°C, being significantly lower at 20°C (see Figure 4c and Table 1). Additionally, seawater *p*CO_2_ appeared to have a stronger effect on mean [HCO_3_^−^]_cf_ than seawater temperature (see *F*s in Table 1).

## 4 DISCUSSION

The analyses and interpretation of average responses in populations and species have proved a powerful and useful tool in advancing our understanding of biological systems and their responses to environmental changes. However, the success of this approach has in some ways eclipsed the ecological and evolutionary significance of individual responses and individual variation (Darwin, 1859). Here, we show that integrating information on inter‐individual variation in the first (apparent) dissociation constant for sea urchin coelomic fluid is key to understanding the nature of and pronounced variability in the acid–base responses of sea urchins *P. lividus* to changes in seawater temperature and *p*CO_2_. Furthermore, we demonstrate that using inter‐individual values for the first (apparent) dissociation constant of the coelomic fluid paint, a significantly different picture in predicting how the acid–base status of urchins will be impacted by future ocean warming (OW) and acidification (OA), compared with the common practice of using mean values for the first (apparent) dissociation constant. Below we discuss these findings are greater detail and discuss the importance of using an individual‐based approach in interpreting species’ acid–base responses to the future environmental challenges of the global change.

Here, we detected a high level of phenotypic variation in the pKʹ_1cf_, values of individual sea urchins. Values ranged between 5.50 at 15°C and 7.51 at 10°C, varying by 36.55% overall, and by 23.32%, 23.10%, and 32.55% at 10, 15, and 20°C, respectively. Not taking such variation into account alters the values of acid–base variables calculated using this “constant” and so changes the outcome of any study of the effects of altered environmental factors on extracellular acid–base balance. Using a mean value for the first (apparent) dissociation constant to calculate coelomic fluid *p*CO_2_ in individuals exposed to combined seawater *p*CO_2_ and temperature leads to an underestimate of the values. When individual variation in the first (apparent) dissociation constant is taken into account when calculating coelomic fluid *p*CO_2_, the outcome of the experiment was different. No significant interaction between seawater *p*CO_2_ and temperature was detected, and seawater temperature had a strong negative effect on urchins’ coelomic fluid *p*CO_2_, especially for the urchins kept at 20°C.

The integration of inter‐individual information also improves the overall predictability of statistical models: for example, *R*_corr_^2^ = 0.449 when using individual pKʹ_1cf_ for *P. lividus *for the effect of elevated seawater temperature and *p*CO_2_ on coelomic fluid [HCO_3_^−^], while *R*_corr_^2^ = 0.395 when using mean pKʹ_1cf _for *P. lividus* to determine the same parameter. The mean pKʹ_1cf_s for extracellular fluids in the crab *Carcinus maenas* and the urchin *P. lividus* are very similar: for *C. maenas*: 6.057, 6.029, and 6.000 at 10, 15, and 20°C, respectively (Truchot, 1976) and *P. lividus*: 6.455, 6.256, and 6.158 at 10, 15, and 20°C, respectively. The result of this similarity is that using mean pKʹ_1cf _for the decapod crab *Carcinus maenas* to calculate acid–base parameters for urchins was not considerably different from using the mean pKʹ_1cf_ for the sea urchin *P. lividus*.

As hypometabolic and osmoconformers echinoids are considered to be particularly vulnerable to climate and global change drivers (see Dupont & Thorndyke, 2013), as also evidenced by a growing number of studies investigating the effects of OW and OA on urchin's acid–base status (e.g., Miles et al., 2007, Spicer et al., 2011; Spicer & Widdicombe, 2012), calcification (e.g., Stumpp, Trübenbach, et al., 2012), growth (e.g., Albright et al., 2012), fecundity and development (e.g., Dupont, Ortega‐Martinez, & Thorndyke, 2010; Byrne, 2011; Byrne et al., 2011), energy budget (e.g. Stumpp, Trübenbach, et al., 2012) and distribution (e.g., Calosi, Rastrick, et al., 2013). Despite this growing attention, still little is known about combined effects of OA and OW on their physiology. Catarino, Bauwens, and Dubois (2012) suggested that seawater *p*CO_2_ had a greater effect than seawater temperature, on *P. lividus* coelomic acid–base status showing that this species is eurythermal. Consequently, this species inhabits the thermally labile intertidal (Ulbricht and Pritchard, 1972; Lawrence, 1990) as it is able to cope with a broad range of temperature conditions before its acid–base status is significantly disrupted. As shown, *P. lividus*, in common with other echinoids (e.g., Spicer et al., 2011; Spicer & Widdicombe, 2012; Stumpp, Trübenbach, et al., 2012; Catarino et al., 2012), develop an extracellular acidosis when seawater *p*CO_2_ increases. This results to some extent in the increase of bicarbonate ions concentration ([HCO3^−^]) (see Farmanfarmaian, 1966; Miles et al., 2007; Stumpp, Trübenbach, et al., 2012; Collard et al., 2013; Collard, Ridder, David, Dehairs, & Dubois, 2015). However, the inter‐individuals approach we adopted revealed a pronounced effect of temperature on the acid–base status of *P. lividus* as individuals’ physiological parameters were all positively affected in urchins exposed at 20°C. Coelomic fluid pH was greater in urchins exposed to elevated seawater *p*CO_2_ and temperatures, suggesting that urchins exposed to future OW and OA may be able to trigger buffering mechanisms to compensate for body fluid acidosis caused by an increase in seawater *p*CO_2_, future studies which investigate longer term exposure and further improving our experimental design will help to further validate our findings, hopefully overcoming current limitations. In our study, increasing the temperature led to an overcompensation of the acidosis incurred. This may be in part explained by the fact that equilibrium constants (pK) of chemical reactions are generally temperature‐dependent, including those for the protonation of imidazole groups (pKIm), imidazole being largely responsible for intracellular and extracellular non‐bicarbonate buffering in ectotherms (Burton, 2002). However, it is important to note that the “imidazole alphastat hypothesis” assumes a single temperature‐dependent pK value for all non‐bicarbonate buffers (Burton, 2002), while we clearly show here that pK values vary considerably among individuals of the same species maintained at the same environmental temperature.

Although the importance of using an individual approach in a global change context has been recently recognized (e.g., Pistevos et al., 2011; Schlegel et al., 2012), studies explicitly addressing this issue at the physiological level are scarce (e.g., Calosi, Turner, et al., 2013; Melatunan et al., 2013). Nonetheless, our study shows that using an individual‐based approach results in a reduced variation in the calculation of sea urchins’ coelomic fluid *p*CO_2_ and [HCO_3_^−^] exposed at 10°C at both ambient and elevated *p*CO_2_ seawater conditions, but a greater variation in urchins’ coelomic fluid *p*CO_2_ for urchins that experienced ambient seawater *p*CO_2_ at 20°C. This finding supports the idea that our understanding of sea urchins’ acid–base regulation, both in general and specifically in responses under global change challenges, is shaped and dependent on the experimental approach used. As a consequence, considering individuals’ variation is fundamental. This is particularly true, as up to now physiological ecologists have largely ignored physiological inter‐individual variation, as they did not perceive it as biologically significant (Bennett, 1987). Trying to raise awareness on the importance of inter‐individual variation, Forsman and Wennersten (2016) produced a detailed review on the experimental and comparative studies which showed how higher levels of inter‐individual variability relate to a reduction in the risk of extinction of species and populations. In light of their study, it will be interesting to investigate the relevance of physiological inter‐individual variation (including acid–base responses as investigated here), and its genetic basis, as a way of elucidating its ecological and evolutionary implications within the context of the global change.

This study is the first, to our knowledge, that uses an individual‐based approach to determine the acid–base responses of an echinoid species facing a multiple factorial global change challenge. As previous studies have taken a mean approach to investigate the same or similar questions, it is difficult to assess whether the interpretation of organisms’ acid–base responses in a changing environment to date is fully accurate. It is possible that previous work determining echinoids sensitivity to the global change depicts at worst an erroneous at best incomplete, picture, particularly if we consider the high complexity of physiological responses. This means that currently, without considering individual responses, it may be very difficult to predict how populations, species, communities, and ecosystems will actually respond to a changing ocean.

## CONFLICT OF INTERESTS

The authors declare no competing or financial interests.

## AUTHOR CONTRIBUTIONS

EG and PC conceived the study. EG conducted the experiment, carried out the biological measurements, calculations, and statistical analyses, with training from PC and JIS. EG wrote the first draft of the manuscript with input from PC. All authors contributed to the final version of the manuscript.

## Data Availability

The full dataset is available on the open access data repository PANGEA. https://doi.pangaea.de/10.1594/PANGAEA.898654
