# Supplementary material for: The importance of inter‐individual variation in predicting species' responses to global change drivers
Source: Ecol Evol. 2019 Mar 28;9(8):4327–39. doi: 10.1002/ece3.4810 (PMC6476784; doi:10.1002/ece3.4810)

Supplementary Information to ‘**The importance of inter-individual variation in predicting species’ responses to global change drivers**’

**List of Authors**: Ella Guscelli^1,2^, John I. Spicer^2^, Piero Calosi^1,2,*^,

^1^ Département de Biologie, Chimie et Géographie, Université du Québec à Rimouski, 300 Allée des Ursulines, Rimouski, Québec, G5L 3A1, Canada.

^2^ Marine Biology and Ecology Research Centre, School of Biological and Marine Sciences, University of Plymouth, Drake Circus, Plymouth, Devon, PL4 8AA, UK.

* Corresponding Author: Ella Guscelli [ella.guscelli@uqar.ca](mailto:ella.guscelli@uqar.ca%20) ; Tel. 001 (418) 509-1513

**Running head**: Inter-individual variation and global changes

**Supporting information**

**Appendix**

**Details on the environmental monitoring and carbonate system characterisation**

Seawater parameters, with the exception of total alkalinity (TA) were measured every day: salinity with a refractometer (D-D The Aquarium Solution Ltd, Essex, UK), oxygen with hand held oxygen meter (Pro2030 Field Dissolved oxygen / Conductivity, YSI Inc., Yellow Springs, OH, USA), pH with a table pH probe (InLab 413, Mettler Toledo, Columbus, OH, USA) connected to a calibrated pH meter (FiveEasy, Mettler Toledo), and temperature with a mercury thermometer. Calibration of the pH probes was carried out using a Tris pH standard following The Guide to Best Practices (Riebesell et al. 2011). Total alkalinity was determined on poisoned samples (0.02 % Mercuric chloride (HgCl_2_)) three times during the duration of the experiment (day 1, 4 and 7) using an Alkalinity Tritator (AS-ALK2, Apollo SciTech Inc., Bogart, GA, USA), coupled with a pH probe (Orion 8102BNUWP ROSS Ultra, Thermo Fisher Scientific Inc., Waltham, MA, USA) and a pH meter (Thermo scientific Orion Star Meter, Thermo Fisher Scientific Inc.). Seawater *p*CO_2_, dissolved Inorganic carbon (DIC), bicarbonate and carbonate ion concentration ([HCO_3_^-^] and [CO_3_^2-^]), as well as calcite and aragonite saturation states (Ω_calc_ and Ω_ara_), were calculated using ‘CO_2_calc’ (Hansen and Robbins, Saint Petersburg, USA) with Mehrbach (1973) constants. Nitrate, nitrite and ammonia tests were also performed bi-weekly with an aquarist test kit (API Saltwater Master Test Kit, Mars Fishcare North America Inc., Chalfont, PA, USA) to ensure the water changes were adequate. Seawater parameters are reported in Table 1S.

**Table S1:** **Seawater physico-chemical parameters (±1 s.e.m.) measured for each experimental treatment. Dissolved inorganic carbon (DIC), carbon dioxide partial pressure (*p*CO_2_), bicarbonate and carbonate ion concentration ([HCO_3_^-^] and [CO_3_^2−^]), calcite and aragonite saturation state (Ω_cal_ and Ω_ara_) were calculated from pH and total alkalinity (TA) using ‘CO_2_calc’ with constants dissociations form Mehrbach et al. (1973).**

|  |  | Control | Elevated *p*CO_2_ | Elevated temperature | Elevated temperature and *p*CO_2_ | Extreme temperature | Extreme temperature and elevated *p*CO_2_ |
| --- | --- | --- | --- | --- | --- | --- | --- |
| Oxygen (DO%) |  | 91.42 ± 1.49 | 89.50 ± 1.71 | 89.48 ± 1.16 | 89.68 ± 1.40 | 84.17 ± 1.15 | 82.57 ± 1.18 |
| salinity (ppt) |  | 32.85 ± 0.15 | 32.78 ± 0.11 | 33 ± 0.12 | 32.78 ± 0.11 | 33.14 ± 0.15 | 33.14 ± 0.19 |
| Temperature (°C) |  | 10.35 ± 0.19 | 10.50 ± 0.20 | 14.85 ± 0.09 | 14.07 ± 0.07 | 20.71 ± 0.10 | 21.00 |
| pH |  | 7.94 ± 0.01 | 7.63 ± 0.01 | 7.98 ± 0.01 | 7.63 ± 0.01 | 7.99 ± 0.01 | 7.68 ± 0.01 |
| TA (μmol kg^-1^) |  | 2216.28 ± 28.39 | 2199.57 ± 21.08 | 2250.57 ± 30.53 | 2200.00 ± 25.61 | 2198.71 ± 48.93 | 2228.57 ± 62.25 |
| DIC (μmol kg^-1^) |  | 2122.43 ± 26.69 | 2197.71 ± 20.61 | 2120.23 ± 29.00 | 2186.70 ± 24.75 | 2036.07 ± 43.58 | 2174.53 ± 59.16 |
| *p*CO_2_ (μatm) |  | 669.36 ± 13.36 | 1420.40 ± 220.04 | 638.50 ± 14.32 | 1476.77 ± 20.60 | 633.88 ± 6.62 | 1399.86 ± 32.54 |
| [HCO_3_^-^] (μmol kg^-1^) |  | 2011.79 ± 24.90 | 2094.17 ± 19.70 | 1992.40 ± 27.13 | 2082.32 ± 23.60 | 1764.23 ± 136.51 | 2063.33 ± 55.72 |
| [CO_3_^2-^] (μmol kg^-1^) |  | 81.37 ± 2.04 | 41.87 ± 0.88 | 103.64 ± 2.27 | 47.00 ± 1.11 | 123.01 ± 4.82 | 66.77 ± 3.28 |
| Ω cal |  | 1.96 ± 0.05 | 1.01 ± 0.02 | 2.50 ± 0.05 | 1.13 ± 0.03 | 2.97 ± 0.12 | 1.62 ± 0.08 |
| Ω ara |  | 1.24 ± 0.03 | 0.63 ± 0.01 | 1.60 ± 0.04 | 0.72 ± 0.02 | 1.93 ± 0.08 | 1.05 ± 0.05 |

**Supplementary Figure Legends**

**Figure S1: The effects of elevated seawater *p*CO_2_ and temperature on inter-individual variation of coelomic fluid (a) pH (pH_cf_) and (b) TCO_2_ (TCO_2cf_) in the sea urchin *Paracentrotus lividus*.** Different temperature treatments are represented by different colours: blue, orange and red for 10, 15 and 20 °C respectively. Seawater *p*CO_2_ ambient (≈ 300 µatm) and elevated (≈ 1000 µatm) are represented by clear and darker colours respectively. Dots represent individual values.

**Figure S2: The effects of elevated seawater *p*CO_2_ and temperature on inter-individual variation of coelomic fluid *p*CO_2_ in the sea urchin *P. lividus*. *p*CO_2cf_ was determined (a) using individual data for *P. lividus*, (b) using the mean individual pK’_1_ for *P. lividus* and (c) using pK’_1_ for *Carcinus maenas* .** Different temperature treatments are represented by different colours: blue, orange and red for 10, 15 and 20 °C respectively. Seawater *p*CO_2_ ambient (≈ 300 µatm) and elevated (≈ 1000 µatm) are represented by clear and darker colours respectively. Dots represent individual values.

**Figure S3: The effects of elevated seawater *p*CO_2_ and temperature on inter-individual variation of coelomic fluid [HCO_3_^-^] in the sea urchin *P. lividus*. [HCO_3_^-^]_cf_ was determined (a) using individual data for *P. lividus*, (b) using the mean individual pK’_1_ for *P. lividus* and (c) using pK’_1_ for *C. maenas*.** Different temperature treatments are represented by different colours: blue, orange and red for 10, 15 and 20 °C respectively. Seawater *p*CO_2_ ambient (≈ 300 µatm) and elevated (≈ 1000 µatm) are represented by clear and darker colours respectively. Dots represent individual values.

**Figure S4: Unpublished data reporting repeated temporal measurements for acid-base levels sampling in *Stronglyocentrotus droebachiensis* (15 °C) exposed to pH= 7.6 for 30 days.** Values are for (a) perivisceral fluid pH and (b) extracellular bicarbonate, from individual urchins sampled sequentially at 0, 6 and 30 days of exposure.

Fi**gure S1**


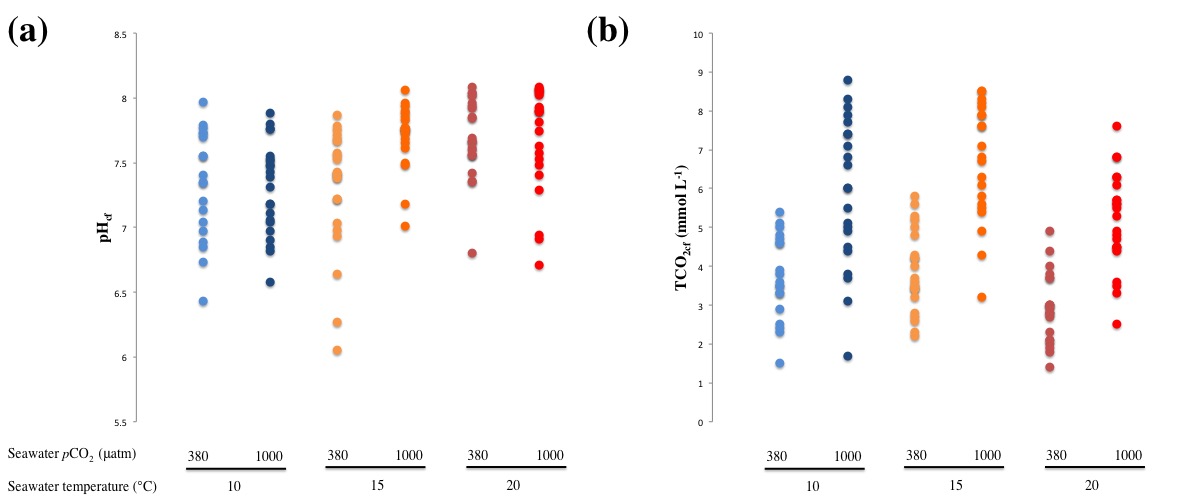


**Figure S2**

**
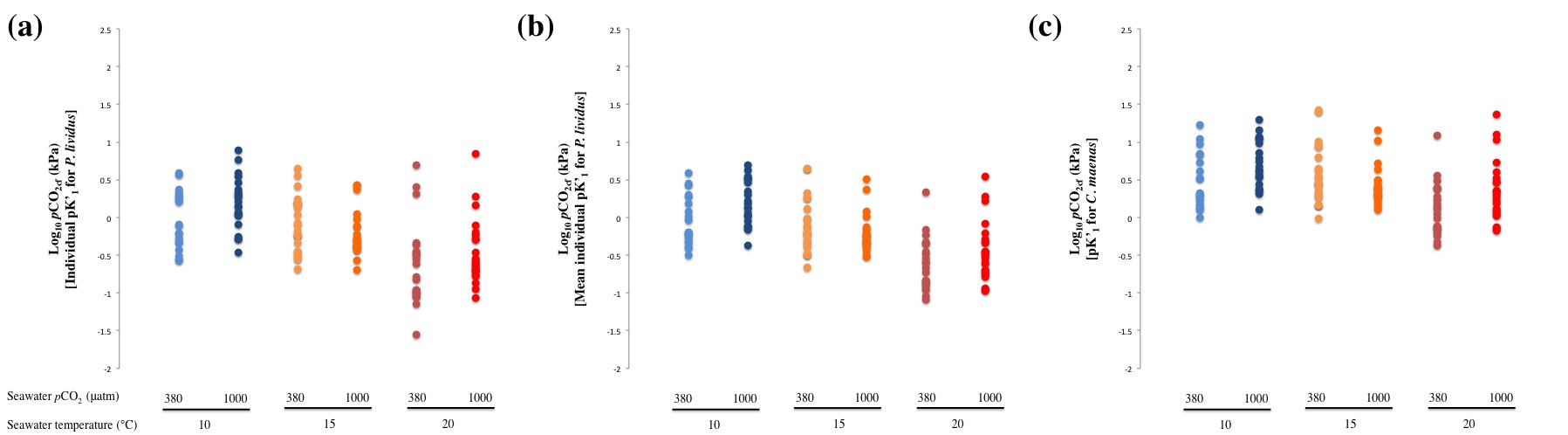
**

**Figure S3**

**
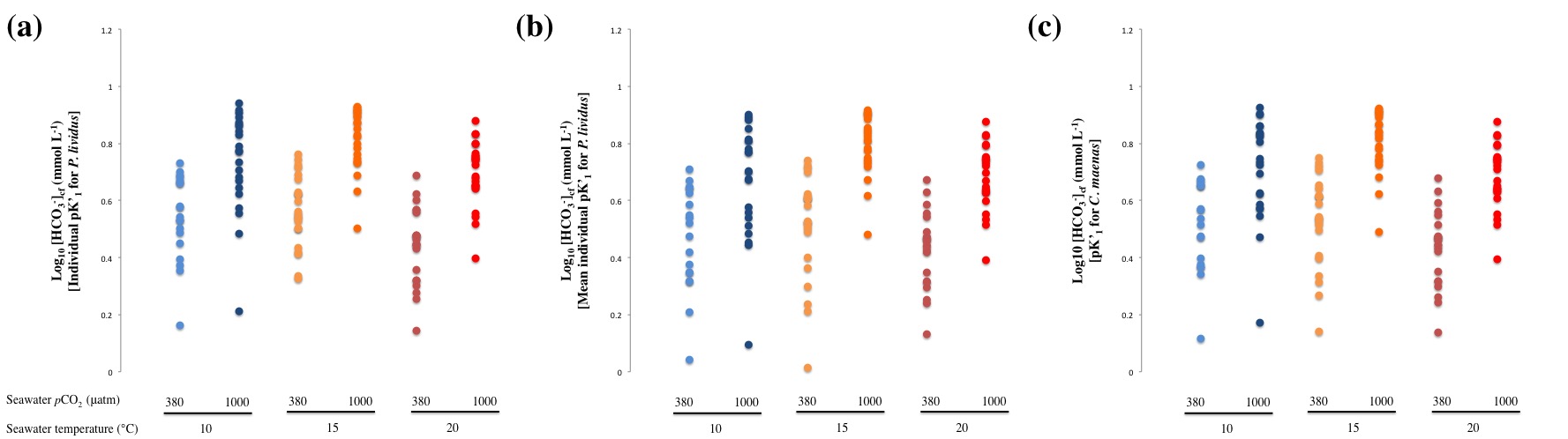
**

**Figure S4**


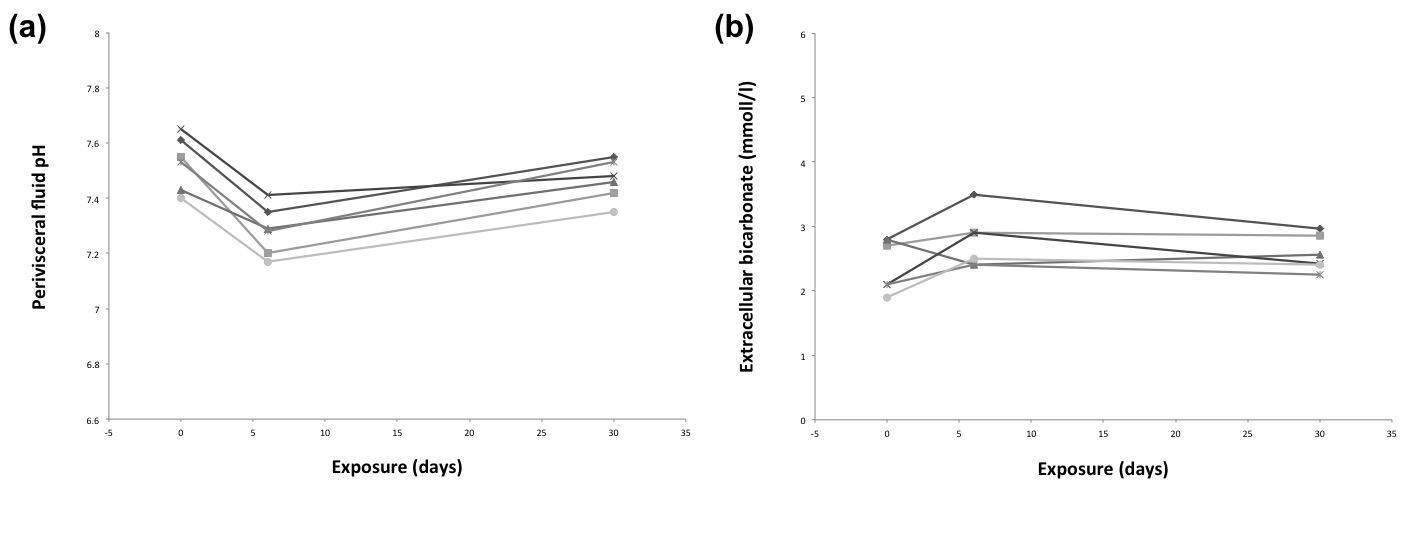

Supplement: Supplementary file 1 [file ECE3-9-4327-s001.docx]
